# A Correlation Study of the Colorectal Cancer Statistics and Economic Indicators in Selected Balkan Countries

**DOI:** 10.3389/fpubh.2020.00029

**Published:** 2020-02-18

**Authors:** Berislav Vekic, Viktorija Dragojevic-Simic, Mihajlo Jakovljevic, Marko Kalezic, Zagor Zagorac, Sasa Dragovic, Rastko Zivic, Filip Pilipovic, Radoje Simic, Dejan Jovanovic, Jovana Milovanovic, Nemanja Rancic

**Affiliations:** ^1^Department of Surgery, Clinical Centre Dr. Dragisa Misovic, Belgrade, Serbia; ^2^Faculty of Medical Sciences, University of Kragujevac, Kragujevac, Serbia; ^3^Centre for Clinical Pharmacology, Military Medical Academy, Belgrade, Serbia; ^4^Medical Faculty of Military Medical Academy, University of Defence, Belgrade, Serbia; ^5^Department of Global Health Economics and Policy, Faculty of Medical Sciences, University of Kragujevac, Kragujevac, Serbia; ^6^Institute of Comparative Economics, Hosei University, Tokyo, Japan; ^7^N.A. Semashko Department of Public Health and Healthcare, I.M. Sechenov First Moscow State Medical University (Sechenov University), Moscow, Russia; ^8^Clinic for General Surgery, Military Medical Academy, Belgrade, Serbia; ^9^Faculty of Medicine, University of Belgrade, Belgrade, Serbia; ^10^Institute for Orthopedic and Surgical Diseases “Banjica”, Belgrade, Serbia; ^11^Department for Plastic Surgery, Institute for Mother and Child Health Care of Serbia Dr. Vukan Cupic, Belgrade, Serbia; ^12^Institute of Radiology, Military Medical Academy, Belgrade, Serbia; ^13^National Health Insurance Fund, Belgrade, Serbia

**Keywords:** non-communicable diseases, colorectal cancer, Balkan region, economic indicators, epidemiological indicators

## Abstract

Colorectal cancer (CRC) is one of the most commonly diagnosed malignant neoplasms. The aim of the study was to evaluate and correlate most important epidemiological and economic indicators of CRC in 11 selected Balkan countries. The number of new CRC cases was 56,960, and the highest 5-year CRC prevalence was in Slovenia, Croatia, and Greece. Age-standardized CRC incidence rates were highest in Slovenia, Serbia, and Croatia, and age-standardized mortality rates were highest in Croatia, Serbia, and Bulgaria. Current Health Expenditure as % of Gross Domestic Product was the highest in Bosnia and Herzegovina and Serbia. The GDP per capita levels have shown positive correlation with the CRC incidence rate and prevalence. Absolute numbers of new and death-related CRC cases and 5-year prevalence in absolute numbers have shown strong positive correlation with GDP in million current US$. It has been shown that various economic indicators can be linked to the rate of incidence and prevalence of the CRC patients in the selected Balkan countries. Therefore, economic factors can influence the epidemiology of CRC, and heavy CRC burden in the Balkan region may be one of the indexes of the economic development.

## Introduction

Non-communicable diseases are responsible for the majority of global deaths, and cancer is expected to be the leading cause of death and the most important barrier to an increasing life expectancy in the twenty-first century (1). Colorectal cancer (CRC; combined cancers of the colon, rectum, and anus) is one of the most common diagnosed malignant neoplasms, considered to be the third among all cancers in terms of incidence, after lung and breast cancer, as well as on the second place in terms of mortality, after lung cancer (2). In 2018, 1,849,518 (10.2%) newly diagnosed CRC cases out of total 18,078,957 newly diagnosed all cancer cases, and 880,792 (9.2%) CRC related deaths out of total 9,555,027 cases of all cancer related deaths, are expected to occur worldwide (2). The highest rates are recorded in Australia and New Zealand, and the lowest estimated one in Western Africa.

The 5-year prevalence for CRC expected worldwide is 4,789,635 cases (2). Age-standardized incidence rates are 23.6 in male and 16.3 per 100,000 in female, also expected to occur worldwide. On the other hand, age-standardized incidence and mortality rates which are expected to occur worldwide are 19.7 and 8.9 per 100,000, respectively.

According to the global trend, incidence rate of CRC is constantly increasing, and the number of CRC cases is expected to increase by 60% until 2030 (more than 2.2 million new cases and 1.1 million deaths) (3, 4).

The CRC 5-year survival rate, for example in the United States, was estimated from a 90% cancer cases detected at the localized stage, 70% for regionalized cancers, to 13% for cases diagnosed with distant metastatic cancer (5).

Health expenditures put more and more pressure on public budgets in most of the countries, especially in connection with the occurrence of population aging throughout the entire Northern Hemisphere (6, 7). They are even more challenging for most Balkan countries due to the economic crisis in Balkan populations and prior civil war for most of them (8). As the incidence of CRC increases constantly, the medical costs of treating these patients are also in the constant rise. Very few papers on CRC status in the Balkan countries exist, especially concerning health care expenditure. The most of them are dealing with the costs of treatment and success of various therapeutic modalities, mostly in Serbia (9–12).

The aim of the study was to evaluate and correlate most important epidemiological and economic indicators of CRC in 11 selected Balkan countries since realistic estimates are crucial to ensure an adequate return on investment in CRC care.

## Materials and Methods

In our study number of new CRC cases was analyzed, number of death related with CRC, as well as 5-year prevalence per 100,000. Also, age-standardized incidence and mortality rates were analyzed. Age-standardized incidence and mortality rates (ASR) are a summary measure of the rate of disease that a population would have if it had a standard age structure. These data are downloaded from the World Health Organization database—GLOBOCAN (https://gco.iarc.fr/).

Selected indicators of health spending analyzed were: Gross Domestic Product (GDP) in million current US$), Gross Domestic Product per Capita in US$; Current Health Expenditure (CHE) as % of Gross Domestic Product (GDP); and Current Health Expenditure (CHE) per Capita in US$. These data are downloaded from the World Health Organization database (https://apps.who.int/nha/database/ViewData/Indicators/en).

Conducted study concerns 11 countries, which belong to the Balkan region, either geographically, or politically: Albania, Bosnia and Herzegovina, Bulgaria, Croatia, Greece, Montenegro, Romania, Serbia, Slovenia, The Republic of North Macedonia, and Turkey (countries are entirely or mostly/partially within the Balkan region).

Statistical analysis was conducted using IBM SPSS Statistics 19.0 computer program (IBM, USA, 2011). Variables were described in the form of absolute number (%). Relationship between variables was tested by Spearman's rank correlation coefficient. All the analyses were evaluated at the level of statistical significance of *p* < 0.05.

The principles of ICH Good Clinical Practice were strictly followed and the approval from the Ethics Committee was obtained (Approval No 26/04/17 for the study protocol No MFVMA/12/17-19, entitled: Cost-effectiveness and cost-utility analysis of CRC treatment and budget impact analysis from the perspective of the patient, hospital, and third-party payer).

## Results

### Cancer Statistics in Balkan Countries

Table 1 summarizes the estimated numbers of new CRC cases and CRC deaths in 11 selected Balkan countries in 2018. In the total population of selected Balkan countries of 143,836,740 citizens, number of new cases of CRC was 56,960. The highest number of new CRC cases was in Turkey, Romania and Greece. Total number of CRC deaths was 30,166. The highest number of CRC deaths was also present in these countries. However, the highest percentage of the new CRC cases in comparison to other cancer location was in Slovenia, Croatia, and Romania, while the highest percentage of CRC death cases comparing with other cancer sites was in Croatia, Bulgaria, and Romania. Therefore, Turkey had the highest number of new CRC cases, as well as CRC deaths in selected Balkan countries in 2018, taking also into account that it has the largest total population. Croatia and Romania had both high percentages of new CRC cases and CRC death cases in comparison to other cancer location when taking into consideration all of the Balkan region.

**Table 1:** Numbers of new colorectal cancer cases and related deaths, age-standardized incidence, and mortality rates (per 100,000) in selected Balkan countries according to GLOBOCAN in 2018 (2).

**Balkan countries**	New cases^*^	Deaths^*^	5-year prevalence^**^	**ASR incidence^**#**^**	**ASR mortality^**#**^**
Albania	374 (4.51)	186 (3.96)	928 (31.63)	8.4	3.7
Bosnia and Herzegovina	1,804 (12.54)	1,074 (11.92)	4,441 (126.75)	26.1	13.3
Bulgaria	4,553 (12.87)	2,687 (14.04)	11,751 (166.99)	28.5	14.9
Croatia	3,356 (13.31)	2,174 (15.06)	8,955 (215.01)	34.1	18.9
Greece	7,117 (10.56)	3,384 (10.16)	19,570 (175.64)	26.2	9.7
Montenegro	208 (8.79)	114 (8.86)	548 (87.09)	18.6	9.2
Romania	10,856 (13.01)	6,155 (12.09)	28,364 (144.86)	26.7	13.7
Serbia	6,049 (12.61)	3,187 (11.84)	15,347 (175.15)	36.7	16.8
Slovenia	1,970 (14.59)	737 (11.52)	5,637 (270.84)	41.1	12.5
The Republic of North Macedonia	984 (12.60)	472 (11.47)	2,429 (116.50)	28.4	12.5
Turkey	19,689 (9.35)	9,996 (8.56)	48,725 (59.48)	21.0	10.2

* Absolute number (% of total number of all new/death cancer site cases);

** Prevalence was shown as absolute number (as proportion of the population per 100,000 persons);

# *Age-standardized (World) incidence and mortality rates*.

Both the lowest number of new CRC cases and the lowest number of CRC deaths in 2018 was registered in Montenegro, as the smallest country in the region.

The highest 5-year CRC prevalence was in Slovenia, Croatia and Greece (270.84; 215.01; 175.64 per 100,000 persons, respectively) (Table 1), while the lowest 5-year prevalence was in Albania.

Age-standardized (World) CRC incidence rates were the highest in Slovenia (41.1), Serbia (36.7), and Croatia (34.1), and age-standardized (World) CRC mortality rates were highest in Croatia (18.9), Serbia (16.8), and Bulgaria (14.9) (Table 1). The lowest values of both of these parameters were registered in Albania.

### Economic and Health CRC Indicators in Balkan Countries

Gross Domestic Product in Balkan countries was the highest in Turkey (863,712 million current US$), Greece and Romania (Table 2). Gross Domestic Product per Capita in US$ was the highest in Slovenia, Greece, and Croatia (more than 10,000 US$). The lowest Gross Domestic Product per Capita was registered in Albania.

**Table 2:** Economic characteristics in selected Balkan countries (2, 13–15).

**Balkan countries**	**Total population**	**GDP (in million current US$)**	**GDP per capita in current US$**	**Current health expenditure as % of GDP**	**Health expenditure per capita in current US$**	**Income level**
Albania	2,934,345	11,864	4,054	6.70	271.54	Upper middle income
Bosnia and Herzegovina	3,503,565	16,910	4,808	9.23	443.78	Upper middle income
Bulgaria	7,036,852	53,238	7,442	8.23	612.48	Upper middle income
Croatia	4,164,772	51,624	12,319	7.18	884.49	High income
Greece	11,142,158	192,691	17,869	8.45	1510.67	High income
Montenegro	629,217	4,845	8,652	6.00	382.10	Upper middle income
Romania	19,580,628	189,005	9,565	4.98	476.37	Upper middle income
Serbia	8,762,022	38,300	5,412	9.14	494.42	Upper middle income
Slovenia	2,081,259	44,709	21,659	8.47	1834.16	High income
The Republic of North Macedonia	2,085,056	10,755	5,168	6.34	327.84	Upper middle income
Turkey	81,916,866	863,712	10,863	4.31	468.65	Upper middle income

*GDP, Gross Domestic Product*.

However, Current Health Expenditure as % Gross Domestic Product was the highest in Bosnia and Herzegovina and Serbia, but the lowest in Turkey. Current Health Expenditure per Capita in US$ was the highest in Slovenia, Greece, and Croatia (1834.16; 1510.67; 884.49, respectively), and the lowest in Albania. Eight countries of 11 have upper middle income according to income level estimated by the World Bank, while three countries have high income, such as Slovenia, Croatia, and Greece (Table 2).

### Correlation Analyses Between Economic and Epidemiological CRC Indicators

In the Balkan countries, the GDP per capita levels (Upper middle income and high income countries) have shown positive correlation with the CRC incidence rate and prevalence (Table 3). The 5-year CRC prevalence per 100,000 persons and GDP per capita level have shown strong positive correlation (5-year prevalence was higher in the high income countries comparing with the Upper middle income ones); *r* = 0.775, *p* = 0.005. Also, ASR CRC incidence rate was higher in the high income countries in comparison to Upper middle income ones; *r* = 0.452, *p* = 0.163. However, ASR CRC mortality rate has not shown any correlations with selected economic CRC indicators (Table 3).

**Table 3:** Correlation analysis between economic and epidemiological colorectal cancer indicators in selected Balkan countries.

	**New cases**	**New cases (% of all new cancer site cases)**	**Deaths**	**Deaths (% of all death cancer site cases)**	**5-year prevalence**	**Prevalence (per 100,000 persons)**	**ASR incidence**	**ASR mortality**	**GDP (in million current US$)**	**GDP per Capita in US$**	**Current health expenditure as % GDP**	**Current health expenditure per capita in US$**
New cases (% of all new cancer site cases)	*r* = 0.291; *p* = 0.385											
Deaths	*r* = 0.991; *p* < 0.001	*r* = 0.236; *p* = 0.484										
Deaths (% of all death cancer site cases)	*r* = 0.236; *p* = 0.484	*r* = 0.809; *p* = 0.003	*r* = 0.255; *p* = 0.450									
5-year prevalence	*r* = 1.000; *p* < 0.001	*r* = 0.291; *p* = 0.385	*r* = 0.991; *p* < 0.001	*r* = 0.236; *p* = 0.484								
Prevalence (per 100,000 persons)	*r* = 0.291; *p* = 0.385	*r* = 0.809; *p* = 0.003	*r* = 0.236; *p* = 0.484	*r* = 0.636; *p* = 0.035	*r* = 0.291; *p* = 0.385							
ASR incidence	*r* = 0.255; *p* = 0.450	*r* = 0.900; *p* < 0.001	*r* = 0.191; *p* = 0.574	*r* = 0.664; *p* = 0.026	*r* = 0.255; *p* = 0.450	*r* = 0.845; *p* = 0.001						
ASR mortality	*r* = 0.364; *p* = 0.270	*r* = 0.779; *p* = 0.005	*r* = 0.378; *p* = 0.252	*r* = 0.916; *p* < 0.001	*r* = 0.364; *p* = 0.270	*r* = 0.588; *p* = 0.057	*r* = 0.747; *p* = 0.008					
GDP (in million current US$)	*r* = 0.927; *p* < 0.001	*r* = 0.273; *p* = 0.417	r = 0.909; *p* < 0.001	*r* = 0.209; *p* = 0.537	*r* = 0.927; *p* < 0.001	*r* = 0.327; *p* = 0.326	*r* = 0.173; *p* = 0.612	*r* = 0.237; *p* = 0.483				
GDP per capita in US$	*r* = 0.482; *p* = 0.133	*r* = 0.464; *p* = 0.151	*r* = 0.400; *p* = 0.223	*r* = 0.145; *p* = 0.670	*r* = 0.482; *p* = 0.133	*r* = 0.645; *p* = 0.032	*r* = 0.400; *p* = 0.223	r = 0.091; p = 0.790	r = 0.609; p = 0.047			
Current health expenditure as % GDP	*r* = −0.127; *p* = 0.709	*r* = 0.291; *p* = 0.385	*r* = −0.109; *p* = 0.750	*r* = 0.345; *p* = 0.298	*r* = −0.127; *p* = 0.709	*r* = 0.573; *p* = 0.066	*r* = 0.464; *p* = 0.151	*r* = 0.333; *p* = 0.318	*r* = −0.136; *p* = 0.689	*r* = −0.082; *p* = 0.811		
Current health expenditure per capita in US$	*r* = 0.518; *p* = 0.102	*r* = 0.691; *p* = 0.019	*r* = 0.455; *p* = 0.160	*r* = 0.491; *p* = 0.125	*r* = 0.518; *p* = 0.102	*r* = 0.918; *p* < 0.001	*r* = 0.709; *p* = 0.015	*r* = 0.451; *p* = 0.164	*r* = 0.609; *p* = 0.047	*r* = 0.809; *p* = 0.003	*r* = 0.418; *p* = 0.201	
Income code	*r* = 0.129; *p* = 0.705	*r* = 0.452; *p* = 0.163	*r* = 0.065; *p* = 0.850	*r* = 0.194; *p* = 0.568	*r* = 0.129; *p* = 0.705	*r* = 0.775; *p* = 0.005	*r* = 0.452; *p* = 0.163	*r* = 0.097; *p* = 0.777	*r* = 0.323; *p* = 0.333	*r* = 0.775; *p* = 0.005	*r* = 0.323; *p* = 0.333	*r* = 0.775; *p* = 0.005

*GDP, Gross Domestic Product; ASR, Age-standardized (World) rates*.

Absolute number of new CRC cases, death-related cases in absolute numbers and 5-year CRC prevalence in absolute numbers have shown strong positive correlation with GDP in million current US$ (these parameters were higher in the countries with higher GDP in comparison to countries with lower GDP); *r* = 0.927, *p* < 0.001; *r* = 0.909, *p* < 0.001; *r* = 0.927, *p* < 0.001, respectively (Table 3).

Five-year CRC prevalence (as proportion of the population per 100,000 persons) was also higher in the countries with higher Current Health Expenditure per Capita in US$ comparing with the countries with lower Current Health Expenditure per Capita in US$); *r* = 0.918, *p* < 0.001 (Table 3).

## Discussion

Colorectal cancer is one of the most common cancers worldwide, with one to two million new cases being diagnosed every year, and with 700,000 cancer-related deaths per year (16). Most cases of CRC are detected in Western countries (55%), but this tendency changes due to the fast development of some countries over the past few years (17). On the other hand, 33% of all CRC-related deaths occurred in Western countries in 2010, due to the improvements made in health systems and the implementation of screening programs (18). The burden of colorectal cancer differs widely across populations, varying with geographical region, age, gender, and socio-economic status (19).

Colorectal cancer incidence and mortality are rapidly growing worldwide (16, 20, 21). Risk for developing CRC is associated with personal features (age, chronic disease history, existence of overweight or obesity, and being tall) or habits (for example, consuming red meat, processed meat, alcoholic drinks, diet poor in folic acid, and vitamin B6), increase the risk for CRC (3, 16, 22). Many of the known risk factors for CRC (age, sedentary lifestyle, Western diet, and smoking) are behaviors traditionally associated with high-income countries (23). Some authors showed that “Western lifestyle” and CRC occurrence had a strong correlation, and that increasing rates of CRC is considered a marker of economic transition (24). However, people living in high-income countries who have a healthy lifestyle have lower CRC risk comparing with the general population in less developed countries.

In recent meta-analyses on CRC risk factors a comprehensive risk modeling strategy in order to predict an individual's risk of developing CRC was developed (25). Inflammatory bowel disease and history of CRC in first-degree relatives were associated with high risk of CRC, while increased body mass index, red meat intake, cigarette smoking, low physical activity, low vegetable consumption, and low fruit consumption were associated with moderately increased risk of CRC. Macrae (26) in the latest update on epidemiology and risk fastors concerning CRC also considers specific genetic disorders, associated with a very high risk of developing colon cancer, such as familial adenomatous polyposis and Lynch syndrome, although together these two conditions account for only ~5% of CRC cases. Ulcerative colitis, Crohn disease and abdominopelvic radiation also significantly increase the risk of subsequent gastrointestinal neoplasms, the majority being CRC. Same author also points out that a large number of clinical, environmental and lifestyle factors often mentioned in the observational studies, such as obesity, diabetes mellitus, taking red and processed meat, tobacco, alcohol, and cholecystectomy are associated with a small and/or uncertain increased risk of CRC (26).

Nevertheless, a modification of lifestyle and/or diet could decrease morbidity, but early detection, such as screening programs (SPs) improves prognosis and reduces mortality (22). Moreover, in the paper concerning CRC SPs in the countries outside the EU-28, which included all the countries we also took into consideration, authors claimed that this health care intervention not only can reduce all types of health care costs, but also decreases the social burden of cancer and protects the most socially endangered members of the society (27).

In the 11 selected Balkan countries, the GDP per capita level (Upper middle income and high income countries), as the best measurement of standard of living in particular country, has shown strong correlation with CRC indicators. The 5-year prevalence was significantly higher in the high income countries, such as Slovenia, Croatia, and Greece comparing to other countries in this region. This especially refer to Albania with the lowest GDP per capita in US$ and lowest 5-year prevalence. Estimation of Current Health Expenditure per capita in the US$ also pointed to Slovenia, Greece and Croatia with highest values, and to Albania with lowest one, in accordance with the fact that this is the indicator of the level of resources channeled to the health relative to other uses. Similarly to our data, in the China, the crude incidence and mortality rates showed positive associations with GDP per capita levels, with high-GDP per capita areas having the highest crude rates, followed by middle- and low-GDP per capita areas (28). The age-standardized incidence rate was highest in the high-GDP per capita areas and lowest in low-GDP per capita areas. In accordance with that this indicator was highest, according to our data, in Slovenia, Serbia, and Croatia. As it was already mentioned, Slovenia and Croatia are High income countries, while Serbia is not. However, Serbia had almost highest Current Health Expenditure as a percentage of GDP in comparison to other Balkan countries in 2018, pointing to the social priority which is given to the health, measured in money resources, including CRC diagnosis. In accordance with this, in the investigation of Altobelli et al. (27) Serbia was included as a country which has population-based SP, implemented despite the crisis and civil war in surroundings, making cancer prevention a priority.

On the contrary, the age-standardized mortality rate was highest in low-GDP per capita areas and lowest in high-GDP per capita areas (28). However, in our study ASR CRC mortality rate has not shown any correlations with selected economic CRC indicators. But when comparison has been made among the selected Balkan countries, ASR mortality rate was the highest in Croatia, High income Balkan country, while Albania as the country with the lowest GDP per Capita in US$ had the lowest value of this indicator. Since our data also showed the lowest values of CRC 5-year prevalence, as well as ASR incidence rate in Albania among the Balkan countries, it can be speculated that insufficient money resources do not allow making a diagnosis in time, as well as monitoring the number of diseased and the deceased patients in an adequate manner. This is somehow supported by the examination of Altobelli et al. (27) who specified that in Albania neither spontaneous nor organized SP was available.

The incidence of CRC worldwide is expected to increase by 80% in the year 2035 (~2.4 million cases). According to data in 2012, 44.6% of the world CRC incidence and 47.8% of its worldwide mortality stems from the Asian continent (19). The Republic of Korea has the highest ASR incidence in the world (ASR incidence is 45). Slovenia, one of our selected Balkan countries is on the 10th place with ASR incidence 37 (19), which is also high according to our data and accounted 41.1 in 2018. It was actually the highest in Balkan region, and it can be explained by the facts that Slovenia is among EU-28 Member states offering organized screening programmes (27). Moreover, among all of these countries, it was one of the 10 which had registered healthcare expenditure (% of GDP) increase concerning period from 2010 to 2014 (29). In other large study which analyzed economic burden of cancer across the European Union, Slovenia spent 72 Euros for health-care costs of cancer per person in 2009, by health-care service categories, including primary care, outpatient care, accident and emergency care, inpatient care and drugs (30). By comparison, European Union average cancer health-care costs amounted 102 Euros. Moreover, according to WHO regions, the EURO region has the highest CRC burden constituting 34.6% of the worldwide incidence and 32.9% of its mortality. Authors showed that in the future an increase in the population and burden of CRC in the Western Pacific Region will be combined, while the stabilization or decrease in ASR for the incidence in the EURO region is foreseen (19). The “Westernization” of many of the West Pacific countries (China, Japan, Korea) contribute for increase of the number of CRC patients, such as increased prevalence of obesity, smoking, high calorie and high meat diets, and sedentary lifestyles. On the other hand, increased incidence of CRC seen over the past few decades in the European countries with subsequent stabilization could be attributed to the adoption of nationwide screening strategies.

The worldwide mortality of CRC is 693,933 with an ASR value of 8.3 per 100,000 in 2012 (19). Asia is still the continent with highest mortality of 331,615. When age is accounted for, Europe is the continent with the highest mortality with an ASR of 12.5 per 100,000, and Hungary is the country with the highest ASR of 20.8 per 100,000. Among WHO regions, mortality is the highest in the EURO region with an ASR of 12.3 per 100,000. According to ASR for mortality, among the highest 10 countries there are several countries from the Balkan region: Croatia on the 2nd place (18.7 ASR mortality), Serbia on the 4th place (16.6), Slovenia on the 6th place (16.2), Bulgaria on the 7th place (16), and Montenegro on the 8th place (15.9). These figures are more or less similar with those obtained in our study for year 2018. The relationship between socio-economic status and CRC mortality is however complex, but generally speaking, the higher CRC mortality, the lower the socioeconomic status of the country was indicated. Actually, limited access to screening in the society with low socio-economic status contributes to a seemingly lower incidence of CRC in these populations with a subsequent increase in mortality, as these patients are presented late in the course of their disease. This is substantiated by the results of Vrdoljak et al. (31) according to which Slovenia, Croatia, Serbia, Bulgaria, and Montenegro, as the Central and Eastern European countries, have higher mortality-to-incidence ratio in comparison to Western European and Nordic countries (Norway, Finland, Sweden, and Denmark) which have higher health expenditure per capita.

Total costs of diagnosis and treatment in the CRC patients are high throughout the world (32–35) and very much depend on the initial TNM stage—the stage I disease is the least costly, whereas stage III is the most expensive due to the high cost of biological agents—monoclonal antibodies (mAbs) (12, 36, 37). In our earlier study, most of the expenses in the end-of-life stage results from inpatient care and administration of chemotherapy (11). In more recent study it was shown that the relative cost of medication was particularly high, accounting from 25.7% (2015) to 30.9% (2017) of total CRC costs in Serbia, while the cost of mAbs dominated other CRC-related expenses and accounted for 11.3, 12.2, 15.0, and 15.2% of all CRC-related expenses in 2014, 2015, 2016, and 2017, respectively (32). High costs associated with the treatment of metastatic CRC, including total drug expenditure was also reported in Croatia (38) and Turkey (39). The scarce data concerning medical costs of cancer services in the Balkan region can, at least partly, be explained by low resources and lack of high-quality cancer registries, national cancer strategic plans, and cost-utility analyses.

To some extent, situation concerning CRC costs associated with the stage of the disease is similar in Western countries with larger GDP. In the German study authors showed that the mean incremental annual costs for each phase of CRC (initial, intermediate, and end-of-life phases), were 26,000; 2,300, and 51,700 euro, respectively (35). In various earlier studies, stage-specific annual cost estimates ranged from 14,982 to 21,264 Euros for early stages and from 29,770 to 34,909 Euros for late stages (40–43).

Therefore, the CRC is an enormous burden worldwide that is expected to increase due to the growth and aging of the population, as well as the adoption of risky behaviors and lifestyle, especially in economically less developed countries (24, 44), such as the Balkan countries. Low socioeconomic status may be an important factor accounting for differences in incidence, mortality and survival rates for CRC. However, better health-care delivery outcomes depend not only on funding: sociocultural, structural, and organizational determinants should be also taken into account when national investment in CRC care are considered and planned (30).

But, globally, ongoing demographic changes will lead to an increasing number of CRC deaths with a doubling of the number of predicted deaths by 2035 (19, 45). Different health systems are facing the challenge of providing care to an increasing population of patients with cancers of all sites, but evidence on costs is limited due to the lack of large longitudinal databases (46). More cost-effective national reimbursement policies could also provide essential savings to the Balkan health systems (12).

## Conclusions

It has been shown that various economic indicators can be linked to the rate of incidence, as well as prevalence of the CRC patients in the Balkan countries. Namely, the GDP per capita levels (Upper middle income and high income countries) have shown positive correlation with the CRC incidence rate and prevalence. Absolute number of new CRC cases and death-related cases, as well as 5-year CRC prevalence in absolute numbers have shown strong positive correlation with GDP in million current US$. Five-year CRC prevalence (as proportion of the population per 100,000 persons) was also higher in the countries with higher Current Health Expenditure per Capita in US$ comparing with the countries with lower Current Health Expenditure per Capita in US$.

Therefore, economic factors can influence the epidemiology of CRC, and heavy CRC burden in the Balkan region may be one of the indexes of the economic development. Moreover, surveillance of CRC occurrence and outcomes for the development of control strategies should be implemented referring to the Balkan regional, as well as local country level in the future, in order to provide savings to the national health systems.

## Data Availability Statement

Publicly available datasets were analyzed in this study. This data can be found here: https://gco.iarc.fr/; https://apps.who.int/nha/database/ViewData/Indicators/en; http://apps.who.int/gho/data/node.main; https://data.worldbank.org/country.

## Author Contributions

BV, NR, MJ, and VD-S jointly designed the study and defined research questions. JM, MJ, FP, and NR did most of the data mining and extraction, purification of files for missing data and artifacts, and statistical analysis. BV, NR, MK, ZZ, DJ, FP, JM, SD, RS, and RZ contributed to the tables and figures creation and interpretation of data. BV, VD-S, MJ, and NR drafted the working version manuscript but all authors contributed to the final version to the extent of important intellectual content.

### Conflict of Interest

The authors declare that the research was conducted in the absence of any commercial or financial relationships that could be construed as a potential conflict of interest. The handling Editor declared a past co-authorship with one of the authors MJ.
